# Trajectories in quality of life of patients with a fracture of the distal radius or ankle using latent class analysis

**DOI:** 10.1007/s11136-017-1670-x

**Published:** 2017-08-01

**Authors:** M. A. C. Van Son, J. De Vries, W. Zijlstra, J. A. Roukema, T. Gosens, M. H. J. Verhofstad, B. L. Den Oudsten

**Affiliations:** 1 CoRPS, Department of Medical and Clinical Psychology, Tilburg University, P.O. Box 90153, 5000 LE Tilburg, The Netherlands; 2 Department of Surgery, Elisabeth-Tweesteden Hospital, Tilburg, The Netherlands; 3 Department of Medical Psychology, Elisabeth-Tweesteden Hospital, Tilburg, The Netherlands; 4 Department of Orthopaedics, Elisabeth-Tweesteden Hospital, Tilburg, The Netherlands; 5 Trauma Research Unit, Department of Surgery, Erasmus MC, University Medical Center Rotterdam, Rotterdam, The Netherlands

**Keywords:** Quality of life, Ankle fractures, Distal radius fractures, Latent class trajectory analyses, Latent class regression model

## Abstract

**Purpose:**

This prospective study aimed to identify the different trajectories of quality of life (QOL) in patients with distal radius fractures (DRF) and ankle fractures (AF). Secondly, it was examined if subgroups could be characterized by sociodemographic, clinical, and psychological variables.

**Methods:**

Patients (*n* = 543) completed the World Health Organization Quality of Life assessment instrument-Bref (WHOQOL-Bref), the pain, coping, and cognitions questionnaire, NEO-five factor inventory (neuroticism and extraversion), and the state-trait anxiety inventory (short version) a few days after fracture (i.e., pre-injury QOL reported). The WHOQOL-Bref was also completed at three, six, and 12 months post-fracture. Latent class trajectory analysis (i.e., regression model) including the Step 3 method was performed in Latent Gold 5.0.

**Results:**

The number of classes ranged from three to five for the WHOQOL-Bref facet and the four domains with a total variance explained ranging from 71.6 to 79.4%. Sex was only significant for physical and psychological QOL (*p* < 0.05), whereas age showed significance for overall, physical, psychological, and environmental QOL (*p* < 0.05). Type of treatment or fracture type was not significant (*p* > 0.05). Percentages of chronic comorbidities were 1.8 (i.e., social QOL) to 4.5 (i.e., physical QOL) higher in the lowest compared to the highest QOL classes. Trait anxiety, neuroticism, extraversion, pain catastrophizing, and internal pain locus of control were significantly different between QOL trajectories (*p* < 0.05).

**Conclusions:**

The importance of a biopsychosocial model in trauma care was confirmed. The different courses of QOL after fracture were defined by several sociodemographic and clinical variables as well as psychological characteristics. Based on the identified characteristics, patients at risk for lower QOL may be recognized earlier by health care providers offering opportunities for monitoring and intervention.

## Introduction

Trauma leading to fractures of the distal radius (DRF) or ankle (AF) is quite common, with incidence rates of 26–32 per 10^4^ person years for DRF [1–3] and 10.1 per 10^4^ person years regarding AF [4]. Patients may experience secondary fracture displacement [5–7], suffer from pain, stiffness, sleep difficulties, reduced grip strength, and/or restricted range of motion [8–12], which affect employment [13–15], sports [8, 9], and quality of life (QOL; i.e., patients’ subjective evaluations of their functioning and well-being [16–18]).

The course of QOL post-fracture may be influenced by sociodemographic, clinical, and psychosocial variables. Some sociodemographic and clinical predictors of health status (HS) [19] and health-related quality of life (HRQOL) [20], constructs related to QOL, have been studied in patients with DRF or AF. However, results were inconclusive with regard to age, sex, educational level, marital status, arthritis, type of treatment, type of fracture, and certain radiographic indices [21, 22]. Moreover, personality and patients’ health beliefs have not been examined in relation to QOL in patients with DRF or AF, although personality traits have shown to be valuable predictors in areas as chronic pain in orthopedics and in oncology research [23–26]. Pain catastrophizing which includes negative pain-related cognitions like rumination, helplessness, and magnification [27] was a significant predictor of HS/HRQOL five to eight months after musculoskeletal trauma [28, 29]. In patients after whiplash injury, the early use of passive pain coping strategies was related to slower recovery [30] and in oncological studies a high level of avoidance coping was associated with impaired HRQOL [31] suggesting the importance of psychological characteristics. Furthermore, health locus of control (HLOC) beliefs, the belief to be either in charge of yourself regarding your health (i.e., internal HLOC) or the externalization of this control to powerful others such as physicians or fate [32, 33], may also be interesting to take into consideration in relation to QOL in patients with fractures. In elderly women with hip fractures, high levels of internal HLOC predicted higher levels of daily living activities [34]. In our study, we will focus on a specific facet of HLOC i.e., pain locus of control (PLOC) [33], which is assumed to be particularly relevant in patients with fractures.

Better insight in the factors that may influence the course of QOL after fracture facilitates the identification of patients that need additional monitoring or care in clinical practice. Moreover, it may offer directions for the development of psychological interventions to improve patients’ QOL. Therefore, in this study, we identified QOL trajectories of patients with DRF or AF up to 12 months post-fracture (i.e., using latent class trajectory analysis) and examined if subgroups could be characterized by sociodemographic, clinical, and psychological variables.

## Methods

### Patients

Patients were invited to participate in this study with inclusion starting January 2012 at the St. Elisabeth Hospital and September 2012 at the TweeSteden Hospital, Tilburg, The Netherlands (i.e., two locations of the same hospital). Analyses were performed on data from patients included up to November 2014. The main inclusion criteria were the diagnosis of an isolated unilateral DRF or AF which was inflicted by trauma (i.e., no stress fractures) and a minimal age of 18 years old. The diagnosis had to be confirmed by X-ray. Patients with multiple trauma were not included (i.e., additional injuries besides the DRF or AF caused by the traumatic event). Because of the focus on self-report measures in this study, patients were excluded when they were not able to complete the questionnaires themselves (e.g., insufficient knowledge of the Dutch language). The presence of severe psychopathology (e.g., suicidal) or severe physical comorbidity (e.g., lung cancer) were exclusion criteria as well.

### Design

Eligible patients were invited to participate in the study within a few days after their visit to the Emergency Department (i.e., during this visit fracture diagnosis was established) by a member of the research team. Patients provided informed consent before entering the study. Patients were asked to complete self-report measures at the time of diagnosis (Time-0_retrospective_), 3 months post-fracture (Time-1), 6 months post-fracture (Time-2), and 12 months post-fracture (Time-3). The measurement at Time-0_retrospective_ consisted in general of a retrospectively reported pre-injury status by the patient to establish a baseline. Personality, pain beliefs, and pain coping were assessed without pre-injury instruction at baseline. Personality traits are assumed to be stable characteristics over time in a variety of situations. In addition, pain beliefs and coping were answered a few days post-fracture because pain levels were expected to be at their highest levels around that time point. Patients received the self-report measures as paper questionnaire booklets at their home addresses. The local Medical Ethics Committee approved the study.

### Fracture classification

DRF and AF were independently classified by a trauma surgeon/senior trauma resident according to the Müller AO classification of long bones [35] based on the primary X-ray. Initial agreement was 61.9%. Consensus meetings were scheduled in which disagreements were discussed.

### Measures

Age, sex, marital status, educational level, employment status, smoking, chronic comorbidities, type of injury, and type of treatment were collected by a general questionnaire added to the booklet of Time-0_retrospective_.

The World Health Organization Quality of Life assessment instrument-Bref (WHOQOL-Bref) is a 26-item QOL questionnaire encompassing four domains: Physical health, Psychological health, Social relationships, and Environment [36]. Moreover, two items form the facet overall QOL and general health. Items are rated on five-point Likert scales with higher scores indicating better QOL. Psychometric properties of the WHOQOL-Bref are satisfactory in patients with different diseases [37–41].

Pain beliefs and coping were assessed with the 42-item Pain, Coping, and Cognition Questionnaire (PCCL) [33, 42]. The PCCL encompasses four subscales: Catastrophizing, Pain coping, Internal locus of control, and External locus of control. Items of the PCCL are rated on a six-point Likert format. A higher score indicates more catastrophizing, higher variability of pain coping strategies, or a higher internal/external locus of control. The psychometrics of the PCCL was examined in chronic pain patients and was adequate to good [33].

The NEO-five factor inventory (NEO-FFI) is a frequently used personality questionnaire that measures the five personality traits of the Five Factor Model [43–45]: Neuroticism, Extraversion, Agreeableness, Conscientiousness, and Openness to experience. For this study, only the subscales Neuroticism (12 items) and Extraversion (12 items) were completed. Items are responded on five-point Likert scales. Higher scores indicate higher levels of neuroticism or extraversion. The psychometric properties of the NEO-FFI appeared to be sufficient [44].

Trait anxiety was measured by the Trait anxiety subscale (10 items) [46] adapted from the state-trait anxiety inventory [47–49]. Items are answered on four-point Likert scales. Higher scores represent a stronger tendency to experience anxiety across different situations. The 10-item trait scale is a reliable and valid measure [46].

### Statistical analyses

Participants were compared with non-participants performing Chi square tests (i.e., sex, type of fracture, AO classification) and an independent samples *t* test (i.e., age).

Latent class trajectory analysis was performed to determine the number of non-observed classes in the course of QOL using the latent class regression model in Latent GOLD 5.0 [50–52]. Analyses were performed repeatedly for the five dependent variables: Physical health, Psychological health, Social relationships, Environment, and the facet Overall QOL and general health of the WHOQOL-Bref. In case of strong non-normality and less than 20 unique scores in a dependent variable, this variable was analyzed as an ordinal variable in which scores were merged in maximal 10 bins of approximately equal size (i.e., minimal 10% of the cases [52]).

The factor ‘time’ was used in the models as a nominal variable with four time points. No covariates were included in the models. Subsequently, models with one to eight classes were estimated. The optimum number of classes was based on the Bayesian Information Criterion (BIC), which is an indicator of model fit taking complexity of the model into account as well. The model with the number of classes with the lowest BIC was selected. Each patient was assigned a class membership probability for each class. The labeling of the classes is based on the level of each group within the model. The Wald(0) test of the predictor time is a global test indicating if any effect of time is present (i.e., if there is a significant deviation from zero). In addition, the Wald(=) test indicates if this effect of time significantly differs between classes.

The Step 3 method was used to take uncertainty in the prediction of class membership into account to prevent bias [51]. Patients in the different classes (i.e., with consequently a different trajectory of QOL) were compared on sociodemographic (i.e., age, sex, marital status, educational level, employment status), clinical (i.e., smoking, chronic comorbidities, type of fracture, AO classification, type of treatment), and psychological characteristics (i.e., personality traits, coping cognitions, and strategies) using the Step 3 method (i.e., Analysis Dependent). The corrected *p* values of the Wald(0) test using the Step 3 method were presented. A 0.05 level of significance was applied to evaluate statistical significance. To facilitate the interpretability of the outcomes, the number and percentage or the mean and standard deviation were shown in the tables where the class membership was based on the highest class probability. The different trajectories were presented as line figures based on the estimated marginal means (continuous dependent variables) and the class means (ordinal dependent variables).

## Results

In total, 543 patients returned at least one of the questionnaire sets at a given time point. The participation rate was 47.0%. Compared to non-participants, participants were older (i.e., respectively 50.4 versus 57.0 years of age; *p* < 0.001). In addition, participants were more likely to be women (72.4% was female), while 60.5% of the non-participants was female (*p* < 0.001). No differences were found on fracture type (i.e., DRF versus AF) and AO classification (i.e., 23/44A versus 23/44B versus 23/44C) between participants versus non-participants (*p* > 0.05). The characteristics of the total sample are shown in Table 1.

**Table 1:** Patients’ sociodemographic and clinical characteristics as well as QOL

	Total *n* = 543 (100)
Age (years, *n* = 543)	57.0 ± 16.6 (59, 18-97)
Sex (*n* = 543)	
Male	150 (27.6)
Female	393 (72.4)
Marital status (*n* = 517)	
Partner	388 (75.0)
No partner	129 (25.0)
Educational level (*n* = 471)	
Low: high school or less	228 (48.4)
High: additional education after high school	243 (51.6)
Employment (*n* = 531)	
Employed	252 (47.5)
Unemployed	279 (52.5)
Smoking (*n* = 525)	
Yes	83 (15.8)
No	442 (84.2)
Chronic comorbidities (*n* = 524)	
Yes	183 (34.9)
No	341 (65.1)
Type of injury (*n* = 533)	
Traffic	62 (11.6)
Work place	38 (7.1)
Home environment	142 (26.6)
Sports	96 (18.0)
Other	195 (36.6)
Type of fracture (*n* = 543)	
Distal radius fracture	297 (54.7)
Ankle fracture	246 (45.3)
AO classification (*n* = 543)	
23/44A	198 (36.5)
23/44A1	76 (14.0)
23/44A2	64 (11.8)
23/44A3	58 (10.7)
23/44B	164 (30.2)
23/44B1	98 (18.0)
23/44B2	30 (5.5)
23/44B3	36 (6.6)
23/44C	169 (31.1)
23/44C1	47 (8.7)
23/44C2	54 (9.9)
23/44C3	68 (12.5)
Isolated medial malleolus fracture^b^	12 (2.2)
Type of treatment (*n* = 543)	
Operative	159 (29.3)
Non-operative	384 (70.7)
QOL^c^	
Overall QOL and general health (*n* = 458)	8.3 ± 1.3
Physical health (*n* = 456)	16.8 ± 2.5
Psychological health (*n* = 457)	15.8 ± 2.2
Social relationships (*n* = 460)	16.0 ± 2.7
Environment (*n* = 456)	16.8 ± 2.3

All values, except for age and quality of life (mean ± standard deviation with the median followed by the minimum and maximum between parentheses) are given as the number of patients, with the percentage between parentheses. For the calculation of the percentages, missings are not included

*NA* not applicable, *AO classification* Müller AO classification of long bones, *QOL* quality of life

^a^Number 23 indicates the bone segment for distal radius fractures whereas number 44 represents the bone segment for ankle fractures in the AO classification system. The frequencies and percentages for the AO classification system are given for the three main groups as well as the nine subgroups

^b^Type of ankle fracture that could not be fitted properly into the AO classification system

^c^Means and standard deviations of QOL at Time-0_retrospective_

### Trajectories of QOL

**Fig. 1:**
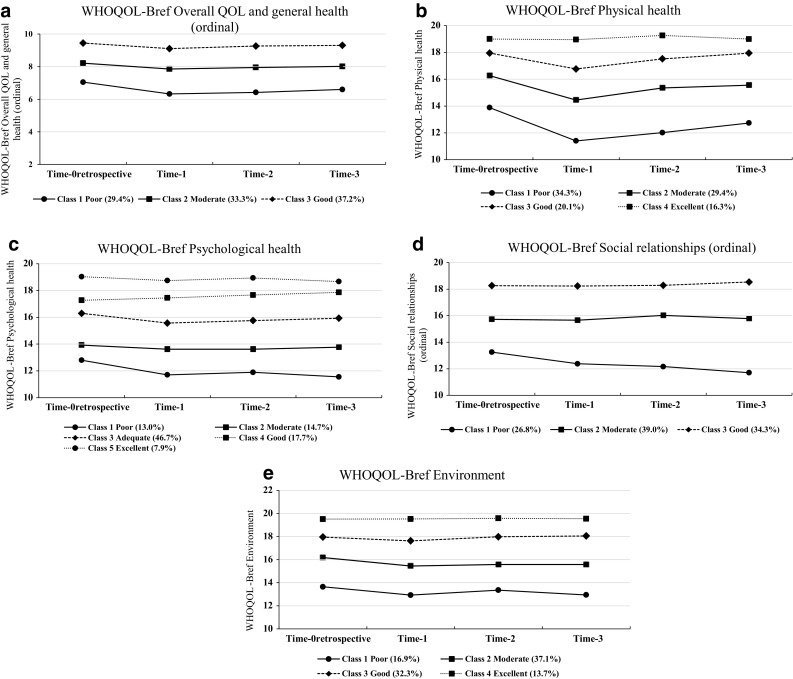
**a** WHOQOL-Bref Overall QOL and general health. *Abbreviations* Time-0_retrospective_ = pre-injury status, Time-1 = 3 months post-fracture, Time-2 = six months post-fracture, Time-3 = 12 months post-fracture, QOL = quality of life, WHOQOL-Bref = World Health Organization Quality of Life assessment instrument-Bref. *Notes* Class means are shown. A higher score indicates a better quality of life. Percentages are shown of the sample included in each class. **b** WHOQOL-Bref Physical health. *Notes* Estimated marginal means are shown. A higher score indicates a better quality of life. Percentages are shown of the sample included in each class. **c** WHOQOL-Bref Psychological health. *Notes* Estimated marginal means are shown. A higher score indicates a better quality of life. Percentages are shown of the sample included in each class. **d** WHOQOL-Bref Social relationships. *Notes* Class means are shown. A higher score indicates a better quality of life. Percentages are shown of the sample included in each class. **e** WHOQOL-Bref Environment. *Notes* Estimated marginal means are shown. A higher score indicates a better quality of life. Percentages are shown of the sample included in each class

Social relationships and Overall QOL and general health were transformed to ordinal variables. The number of classes ranged from three to five (Table 2). The total variance explained by the models ranged from 71.6 to 79.4%. The effects of time were present in all models. The time effect was significantly different between the classes (Fig. 1b–e), except for Overall QOL and general health (Wald(=) *p* = 0.87, Fig. 1a). Table 3 shows the optimum number of classes based on the lowest BIC values for all QOL models.

**Table 2:** Characteristics of the class models of the WHOQOL-Bref

Dependent variable	Analyzed as continuous or ordinal variable	Nr of classes	*R* ^2^	Wald(0)^a^ *p* value	Wald(=)^a^ *p* value
Overall QOL and general health	Ordinal (4 categories)	3	.72	**<0.001**	0.87
Physical health	Continuous	4	.75	**<0.001**	**<0.001**
Psychological health	Continuous	5	.78	**<0.001**	**0.009**
Social relationships	Ordinal (7 categories)	3	.72	**0.023**	**0.01**
Environment	Continuous	4	.79	**<0.001**	**0.004**

Significant *p* values (*p* < 0.05) are marked in bold. The number between brackets in the column ‘Analyzed as continuous or ordinal variable’ represents the number of categories after transformation from a continuous variable to an ordinal variable

*QOL* quality of life, *Nr* number

^a^Wald tests of time effects

**Table 3:** Bayesian information criterion (BIC) values of all models

No of classes	Overall QOL and general health (ordinal: 4 categories)	Physical health	Psychological health	Social relationships (ordinal: 7 categories)	Environment
1	3859,573	6751,418	6353,137	5415,529	6425,771
2	3468,745	6147,457	5837,232	5035,959	5837,804
3	**3412,713**	5982,306	5696,122	**4933,103**	5628,515
4	3414,700	**5946,118**	5672,432	4955,431	**5530,121**
5	3440,841	5957,006	**5665,538**	4988,167	5531,857
6	3473,686	5967,387	5672,167	5021,783	5535,287
7	3513,532	5983,384	5689,382	5064,903	5553,142
8	3544,726	5994,313	5708,427	5117,752	5569,405

The BIC value of the final models are marked in bold

*Overall QOL and general health* included three classes: Poor, Moderate, and Good (Table 4). Sociodemographic factors were significant, except for sex. Patients in the Poor QOL class had the highest age, were less frequently partnered, and had the lowest employment rate. In the Good QOL class, patients were 1.6 times more often highly educated compared to the Poor QOL class. Classes differed significantly on smoking and the presence of chronic comorbidities. The proportion of patients being non-smokers and patients without chronic comorbidities increased per class in ascending magnitude of QOL. All psychological variables reached significance, expect for pain coping. Patients in the Good QOL class had higher mean scores on extraversion and internal PLOC, and lower scores for neuroticism and trait anxiety in this class, compared to the other two classes.

**Table 4:** Sociodemographic, clinical, and psychological characteristics for the three classes on WHOQOL-Bref Overall QOL and general health (ordinal) as well as for the four classes on WHOQOL-Bref Physical health

Characteristics	Overall QOL and general health (ordinal)				Physical health				
	Class 1 Poor (*N* = 158; 29.4%)	Class 2 Moderate (*N* = 179; 33.3%)	Class 3 Good (*N* = 200; 37.2%)	*p* value corrected	Class 1 Poor (*N* = 106; 20.1%)	Class 2 Moderate (*N* = 155; 29.4%)	Class 3 Good (*N* = 181; 34.3%)	Class 4 Excellent (*N* = 86; 16.3%)	*p* value corrected
Sociodemographic characteristics									
Age	60.8 ± 15.1	57.4 ± 16.6	53.4 ± 17.2	**<0.001**	59.6 ± 16.0	60.4 ± 15.9	53.8 ± 16.7	52.4 ± 16.7	**<0.001**
Sex									
Male	37 (23.4)	45 (25.1)	66 (33.0)	0.098	26 (24.5)	29 (18.7)	62 (34.3)	31 (36.0)	**0.013**
Female	121 (76.6)	134 (74.9)	134 (67.0)		80 (75.5)	126 (81.3)	119 (65.7)	55 (64.0)	
Partner									
Yes	95 (66.0)	133 (77.3)	157 (80.1)	**0.014**	66 (67.3)	101 (68.7)	147 (83.1)	68 (81.0)	**0.015**
No	49 (34.0)	39 (22.7)	39 (19.9)		32 (32.7)	46 (31.3)	30 (16.9)	16 (19.0)	
Educational level									
Low education	76 (60.8)	80 (51.6)	70 (37.4)	**<0.001**	55 (65.5)	76 (57.6)	63 (38.2)	27 (33.3)	**<0.001**
High education	49 (39.2)	75 (48.4)	117 (62.6)		26 (34.5)	56 (42.4)	102 (61.8)	54 (66.7)	
Employment									
Yes	55 (36.4)	87 (49.2)	108 (54.5)	**<0.001**	29 (28.4)	67 (44.1)	104 (57.8)	49 (58.3)	**<0.001**
No	96 (63.6)	90 (50.8)	90 (45.5)		73 (71.6)	85 (55.9)	76 (42.2)	35 (41.7)	
Clinical characteristics									
Smoking									
Yes	32 (21.2)	32 (18.5)	18 (9.2)	**0.01**	20 (19.8)	24 (16.1)	30 (16.8)	8 (9.6)	0.43
No	119 (78.8)	141 (81.5)	178 (90.8)		81 (80.2)	125 (83.9)	149 (83.2)	75 (90.4)	
Chronic comorbidities									
Yes	94 (62.7)	55 (31.6)	33 (16.9)	**<0.001**	65 (65.0)	54 (36.2)	47 (26.3)	12 (14.5)	**<0.001**
No	56 (37.3)	119 (68.4)	162 (83.1)		35 (35.0)	95 (63.8)	132 (73.7)	71 (85.5)	
Diagnosis									
Distal radius fracture	92 (58.2)	98 (54.7)	104 (52.0)	0.27	54 (50.9)	92 (59.4)	96 (53.0)	45 (52.3)	0.35
Ankle fracture	66 (41.8)	81 (45.3)	96 (48.0)		52 (49.1)	63 (40.6)	85 (47.0)	41 (47.7)	
AO classification									
Group A	62 (40.0)	65 (37.1)	68 (34.9)	0.64	37 (35.9)	62 (40.5)	64 (36.0)	28 (34.1)	0.99
Group B	46 (29.7)	52 (29.7)	64 (32.8)		34 (33.0)	43 (28.1)	56 (31.5)	28 (34.1)	
Group C	47 (30.3)	58 (33.1)	63 (32.3)		32 (31.1)	48 (31.4)	58 (32.6)	26 (31.7)	
Type of treatment									
Non-operative	116 (73.4)	122 (68.2)	140 (70.0)	0.54	74 (69.8)	111 (71.6)	117 (64.6)	67 (77.9)	0.21
Operative	42 (26.6)	57 (31.8)	60 (30.0)		32 (30.2)	44 (28.4)	64 (35.4)	19 (22.1)	
Psychological characteristics									
Trait anxiety	19.2 ± 5.8	16.3 ± 4.4	13.4 ± 3.2	**<0.001**	19.7 ± 5.8	17.3 ± 5.0	14.3 ± 3.7	12.9 ± 2.8	**<0.001**
Neuroticism	30.9 ± 7.1	27.5 ± 6.7	23.0 ± 6.4	**<0.001**	31.0 ± 7.0	29.3 ± 7.2	25.0 ± 6.25	20.8 ± 5.5	**<0.001**
Extraversion	39.0 ± 6.6	41.7 ± 6.1	44.3 ± 6.2	**<0.001**	39.8 ± 6.8	40.3 ± 6.4	42.5 ± 6.4	46.2 ± 5.1	**<0.001**
Pain catastrophizing	2.5 ± 0.9	2.1 ± 0.7	1.8 ± 0.6	**<0.001**	2.8 ± .9	2.1 ± .7	1.9 ± .6	1.7 ± .6	**<0.001**
Pain coping	3.5 ± 0.9	3.6 ± 0.9	3.6 ± 1.0	0.42	3.5 ± .9	3.6 ± .9	3.6 ± .9	3.4 ± 1.0	0.49
Internal locus of pain control	3.6 ± 1.0	3.8 ± 0.8	4.0 ± 0.9	**<0.001**	3.4 ± 1.0	3.9 ± .8	4.0 ± .9	4.0 ± 1.0	**<0.001**
External locus of pain control	3.0 ± 1.0	2.8 ± 0.9	2.6 ± 0.9	**<0.001**	3.0 ± .9	3.0 ± 1.0	2.7 ± .9	2.5 ± .9	**<0.001**

*Low education* high school or less, *high education* additional education after high school

Values are given as the number of patients, with the percentages in parentheses. For the calculation of the percentages, missing are not included. Mean ± standard deviation are presented for age and the psychological characteristics. *p* values corrected for classification error are extracted from the Step-3 method (dependent) of Latent GOLD. Significant *p* values (*p* < 0.05) are marked in bold

The four trajectories of *Physical health* contained a Poor, Moderate, Good, and Excellent class (Table 4). Significant differences were found on all examined sociodemographic and clinical variables, except chronic comorbidities. Patients in the Good and Excellent QOL class were younger than the patients in the Poor and Moderate QOL class. Female contribution was lowest in the Good and Excellent QOL classes, but still up to 65.7%. Moreover, in the Poor and Moderate QOL class, up to 68.7% had a partner compared to 81.0–83.1% of the patients in the Good and Excellent QOL classes. In the Excellent QOL group, patients had almost twice as often a high educational level and employed compared to the Poor QOL group. Patients in the Poor QOL group had more than fourth as often chronic comorbidities compared to patients in the Excellent QOL group. Classes differed significantly on all the psychological characteristics, except pain coping. Patients in the four different classes, in ascending magnitude of QOL, had lower scores on trait anxiety, neuroticism, and pain catastrophizing, and higher scores on extraversion. Patients in the Good and Excellent QOL class had higher scores on internal PLOC and lower scores on external PLOC compared to the other two classes.

The five trajectories of *Psychological health* included a Poor, Moderate, Adequate, Good, and Excellent class (Table 5). All sociodemographic variables reached significance. Patients in the Moderate QOL class were the oldest. In the Good and Excellent QOL group, the proportion male and partnered patients were higher than in the classes with lower QOL. More than half of the patients in the Adequate, Good, and Excellent QOL class had a high educational level and were more often employed. The proportion of patients reporting chronic comorbidities was lower for those classes representing higher QOL. Pain coping and external PLOC were not significant. Lower scores on trait anxiety, neuroticism, and pain catastrophizing, as well as higher scores on extraversion and internal PLOC were found for those trajectories representing higher QOL in ascending order.

**Table 5:** Sociodemographic, clinical, and psychological characteristics for the five classes on WHOQOL-Bref Psychological health

Characteristics		Psychological health				
	Class 1 Poor (*N* = 69; 13.0%)	Class 2 Moderate (*N* = 78; 14.7%)	Class 3 Adequate (*N* = 248; 46.7%)	Class 4 Good (*N* = 94; 17.7%)	Class 5 Excellent (*N* = 42; 7.9%)	*p* value corrected
Sociodemographic characteristics						
Age	57.4 ± 16.4	62.0 ± 16.5	55.2 ± 16.7	56.0 ± 15.7	57.3 ± 18.3	**0.039**
Sex						
Male	13 (18.8)	13 (16.7)	73 (29.4)	33 (35.1)	15 (35.7)	**0.029**
Female	56 (81.2)	65 (83.3)	175 (70.6)	61 (64.9)	27 (64.3)	
Partner						
Yes	40 (63.5)	50 (68.5)	178 (74.5)	81 (87.1)	34 (82.9)	**0.012**
No	23 (36.5)	23 (31.5)	61 (25.5)	12 (12.9)	7 (17.1)	
Educational level						
Low education	36 (66.7)	36 (57.1)	92 (42.2)	41 (45.1)	17 (45.9)	**0.006**
High education	18 (33.3)	27 (42.9)	126 (57.8)	50 (54.9)	20 (54.1)	
Employment						
Yes	27 (40.3)	26 (33.3)	129 (53.1)	45 (48.4)	22 (53.7)	**0.029**
No	40 (59.7)	52 (66.7)	114 (46.9)	48 (51.6)	19 (46.3)	
Clinical characteristics						
Smoking						
Yes	13 (19.7)	16 (21.1)	36 (14.8)	13 (14.0)	4 (10.5)	0.32
No	53 (80.3)	60 (78.9)	207 (85.2)	80 (86.0)	34 (89.5)	
Chronic comorbidities						
Yes	35 (53.8)	32 (41.6)	86 (35.7)	22 (23.4)	6 (15.8)	**<0.001**
No	30 (46.2)	45 (58.4)	155 (64.3)	72 (76.6)	32 (84.2)	
Diagnosis						
Distal radius fracture	47 (68.1)	47 (60.3)	124 (50.0)	49 (52.1)	23 (54.8)	0.21
Ankle fracture	22 (31.9)	31 (39.7)	124 (50.0)	45 (47.9)	19 (45.2)	
AO classification						
Group A	27 (39.1)	40 (51.9)	85 (35.0)	30 (34.1)	11 (26.2)	0.15
Group B	23 (33.3)	17 (22.1)	80 (32.9)	26 (29.5)	16 (38.1)	
Group C	19 (27.5)	20 (26.0)	78 (32.1)	32 (36.4)	15 (35.7)	
Type of treatment						
Non-operative	52 (75.4)	56 (71.8)	169 (68.1)	66 (70.2)	30 (71.4)	0.56
Operative	17 (24.6)	22 (28.2)	79 (31.9)	28 (29.8)	12 (28.6)	
Psychological characteristics						
Trait anxiety	22 ± 6.1	20.0 ± 4.1	15.1 ± 3.7	13.1 ± 2.9	11.9 ± 2.2	**<0.001**
Neuroticism	34.4 ± 7.2	31.3 ± 5.4	26.3 ± 6.3	22.5 ± 5.1	19.0 ± 6.1	**<0.001**
Extraversion	37.0 ± 6.3	38.8 ± 6.5	42.1 ± 6.2	44.8 ± 4.9	47.4 ± 5.5	**<0.001**
Pain catastrophizing	2.6 ± .9	2.3 ± .7	2.1 ± .7	1.8 ± .7	1.5 ± .5	**<0.001**
Pain coping	3.6 ± .8	3.4 ± .9	3.6 ± 1.0	3.6 ± .9	3.8 ± 1.1	0.44
Internal pain locus of control	3.5 ± .9	3.6 ± .8	3.9 ± .9	4.0 ± .9	4.1 ± 1.1	**<0.001**
External pain locus of control	3.0 ± 1.0	2.8 ± .8	2.8 ± 1.0	2.7 ± .9	2.7 ± .9	0.47

*Notes:* low education = high school or less, high education = additional education after high school

Values are given as the number of patients, with the percentages in parentheses. For the calculation of the percentages, missing are not included. Mean ± standard deviation are presented for age and the psychological characteristics. *p* values corrected for classification error are extracted from the Step-3 method (dependent) of Latent GOLD. Significant *p* values (*p* < 0.05) are marked in bold

The three classes of *Social relationships* contained a Poor, Moderate, and Good class (Table 6). Only two out of five sociodemographic variables were significant: marital status and employment. In the Poor QOL class the proportion of having a partner was lowest. The proportion of patients being employed increased per class in ascending magnitude of QOL. Smoking and chronic comorbidities reached significance. Almost twice as often patients smoked in the Poor QOL class and had chronic comorbidities compared to the Good QOL class. The psychological variables were all significant, except for pain coping and external PLOC. The lowest scores for trait anxiety and neuroticism, and the highest scores on extraversion were detected for the good QOL class. Patients with the strongest tendency to catastrophize regarding pain and using the least internal PLOC were found in the Poor QOL class.

**Table 6:** Sociodemographic, clinical, and psychological characteristics for the three classes on WHOQOL-Bref Social relationships (ordinal) as well as for the four classes on WHOQOL-Bref Environment

Characteristics	Social relationships (ordinal)				Environment				
	Class 1 Poor (*N* = 143; 26.8%)	Class 2 Moderate (*N* = 208; 39.0%)	Class 3 Good (*N* = 183; 34.3%)	*p* value corrected	Class 1 Poor (*N* = 90; 16.9)	Class 2 Moderate (*N* = 197; 37.1%)	Class 3 Good (*N* = 171; 32.3%)	Class 4 Excellent (*N* = 73; 13.7)	*p* value corrected
Sociodemographic characteristics									
Age	60.0 ± 15.8	57.3 ± 16.7	54.3 ± 16.8	0.065	61.8 ± 14.6	55.6 ± 17.2	54.6 ± 16.8	58.1 ± 15.6	**0.005**
Sex									
Male	39 (27.3)	65 (31.3)	45 (24.6)	0.33	22 (24.4)	59 (29.9)	47 (27.5)	21 (28.8)	0.87
Female	104 (72.7)	143 (68.8)	138 (75.4)		68 (75.6)	138 (70.1)	124 (72.5)	52 (71.2)	
Partner									
Yes	86 (64.2)	155 (78.3)	145 (81.5)	**0.004**	49 (59.8)	139 (73.9)	134 (80.2)	62 (87.3)	**<0.001**
No	48 (35.8)	43 (21.7)	33 (18.5)		33 (40.2)	49 (26.1)	33 (19.8)	9 (12.7)	
Educational level									
Low education	64 (57.7)	86 (47.3)	72 (41.9)	0.064	51 (75.0)	94 (55.3)	49 (31.4)	28 (40.6)	**<0.001**
High education	47 (42.3)	96 (52.7)	100 (58.1)		17 (25.0)	76 (44.7)	107 (68.6)	41 (59.4)	
Employment									
Yes	53 (38.4)	98 (48.0)	100 (55.2)	**0.04**	25 (29.1)	95 (49.2)	101 (59.4)	28 (39.4)	**<0.001**
No	85 (61.6)	106 (52.0)	81 (44.8)		61 (70.9)	98 (50.8)	69 (40.6)	43 (60.6)	
Clinical characteristics									
Smoking									
Yes	29 (21.2)	33 (16.6)	21 (11.6)	**.048**	22 (26.2)	29 (15.2)	23 (13.8)	8 (11.1)	**0.015**
No	108 (78.8)	166 (83.4)	160 (88.4)		62 (73.8)	162 (84.8)	144 (86.2)	64 (88.9)	
Chronic comorbidities									
Yes	59 (44.0)	77 (38.3)	44 (24.3)	**0.001**	49 (59.8)	67 (35.1)	48 (28.6)	15 (20.8)	**<0.001**
No	75 (56.0)	124 (61.7)	137 (75.7)		33 (40.2)	124 (64.9)	120 (71.4)	57 (79.2)	
Diagnosis									
Distal radius fracture	81 (56.6)	116 (55.8)	95 (51.9)	0.7	59 (65.6)	100 (50.8)	85 (49.7)	46 (63.0)	0.055
Ankle fracture	62 (43.4)	92 (44.2)	88 (48.1)		31 (34.4)	97 (49.2)	86 (50.3)	27 (37.0)	
AO classification									
Group A	61 (43.3)	71 (34.8)	61 (34.5)	0.27	39 (43.3)	68 (35.6)	62 (37.1)	24 (33.8)	0.68
Group B	40 (28.4)	63 (30.9)	60 (33.9)		26 (28.9)	57 (29.8)	56 (33.5)	23 (32.4)	
Group C	40 (28.4)	70 (34.3)	56 (31.6)		25 (27.8)	66 (34.6)	49 (29.3)	24 (33.8)	
Type of treatment									
Non-operative	107 (74.8)	142 (68.3)	126 (68.9)	0.32	68 (75.6)	132 (67.0)	117 (68.4)	55 (75.3)	0.27
Operative	36 (25.2)	66 (31.7)	57 (31.1)		22 (24.4)	65 (33.0)	54 (31.6)	18 (24.7)	
Psychological characteristics									
Trait anxiety	19.1 ± 5.7	15.7 ± 4.5	13.9 ± 3.7	**<0.001**	20.6 ± 5.3	16.6 ± 5.2	14.3 ± 3.6	13.3 ± 3.1	**<0.001**
Neuroticism	30.7 ± 7.3	26.8 ± 6.4	23.5 ± 7.1	**<0.001**	32.2 ± 6.5	28.1 ± 6.9	24.2 ± 6.8	22.7 ± 6.6	**<0.001**
Extraversion	38.7 ± 6.4	41.0 ± 6.0	45.4 ± 5.9	**<0.001**	39.0 ± 6.7	40.6 ± 6.0	48.8 ± 6.1	44.4 ± 7.3	**<0.001**
Pain catastrophizing	2.3 ± 0.8	2.2 ± 0.7	1.9 ± 0.8	**<0.001**	2.6 ± 0.8	2.2 ± 0.7	2.0 ± 0.7	1.7 ± 0.6	**<0.001**
Pain coping	3.5 ± 0.9	3.6 ± 0.9	3.6 ± 1.0	0.46	3.6 ± 1.0	3.5 ± 0.8	3.7 ± 1.0	3.6 ± 1.1	0.42
Internal pain locus of control	3.6 ± 0.9	3.9 ± 0.9	3.9 ± 0.9	**0.008**	3.5 ± 0.8	3.8 ± 0.9	4.0 ± 0.9	4.0 ± 1.0	**<0.001**
External pain locus of control	2.8 ± 1.1	2.8 ± 0.9	2.7 ± 0.9	0.46	3.3 ± 1.0	2.8 ± 0.9	2.6 ± 0.9	2.5 ± 1.0	**<0.001**

*Low education* high school or less, *high education* additional education after high school

Values are given as the number of patients, with the percentages in parentheses. For the calculation of the percentages, missing are not included. Mean ± standard deviation are presented for age and the psychological characteristics. *p* values corrected for classification error are extracted from the Step-3 method (dependent) of Latent GOLD. Significant *p* values (*p* < 0.05) are marked in bold

The four trajectories of *Environment* encompassed a Poor, Moderate, Good, and Excellent class (Table 6). Classes differed significantly on all sociodemographic variables, except for sex. Patients in the Poor and Excellent QOL classes were older compared with the Moderate and Good QOL classes. In the Poor and Moderate QOL class, up to 73.9% had a partner whereas in the Good and Excellent QOL at least 80.2% reported having a partner. The proportion of patients with high educational level was the highest in the classes Good and Excellent QOL. Additionally, in the classes Good and Moderate QOL patients had most often a job. The proportion of non-smokers and patients without chronic comorbidities increased per class in ascending magnitude of QOL. Trends showed that lower scores on trait anxiety, neuroticism, pain catastrophizing, and external PLOC were observed in the classes in ascending magnitude of QOL. The highest mean scores for extraversion and internal PLOC were found for the Good and Excellent QOL class compared to the other classes.

## Discussion

This was the first study using latent class trajectory analyses to identify QOL trajectories (i.e., classes) in patients with a DRF or AF up to 12 months after fracture. In addition, we explored if these patient groups differed on sociodemographic, clinical, and psychological variables (i.e., biopsychosocial approach). The acquired knowledge can facilitate the identification of patients that might need additional monitoring or care.

Subgroups were characterized by several sociodemographic variables of which clinicians are advised to take notice of when treating patients with AF or DRF: i.e., age, sex, marital status, educational level, and employment status. Prior research on AF and DRF in relation to HS and HRQOL reported mainly inconsistent findings on the role of sociodemographic variables [21, 22]. Generally, we found that patients in the lower QOL trajectories (i.e., overall, physical, and environmental) were older. The proportion of women was higher in the lower physical and psychological QOL trajectories. Two studies in AF and DRF are mainly in agreement with these results suggesting that women are at risk for lower HS after fracture [53, 54]. In addition, patients in the higher QOL trajectories more frequently had a partner, showing protective value of the presence of a significant other. Furthermore, a higher educational level (i.e., except for social QOL) and higher job participation were found in the trajectories representing higher QOL. The positive association of educational level with QOL was also reflected in two studies on DRF and AF that reported lower physical HS in patients with lower formal education [55, 56].

Previous research was inconclusive on the role of clinical variables [21, 22] but our study suggests an important distinction between injury-specific and general clinical variables. The QOL trajectories showed no significant differences on injury-specific variables: fracture diagnosis (DRF versus AF), type of treatment (operative versus non-operative treatment), and AO classification. The finding that diagnosis was not significant could be explained by the use of the WHOQOL-BREF, a generic QOL instrument. This instrument is completed by the patient (subjective), but also contains items about the level of satisfaction (e.g., ‘How much do you enjoy life?’) and to what extent a patient is bothered (subjective), instead of items (e.g., ‘Are you able to walk the stairs) that could be considered objective items, because they could be completed by someone else by observing the patient’s functioning. Only general clinical variables (i.e., chronic comorbidities and smoking) were significantly related to class membership. Percentages of chronic comorbidities were 1.8 (i.e., social QOL) to 4.5 (i.e., physical QOL) higher in the lowest QOL class compared to the highest QOL class.

The importance of personality was confirmed. Patients in the trajectories representing lower QOL (i.e., all QOL domains and the overall facet), had higher trait anxiety and neuroticism scores but lower scores on extraversion. Our results are in line with prior research [23–25]. However, one study [57] found no significant relationship between neuroticism and functional status in DRF assessed with the Disabilities of Arm, Shoulder, and Hand (DASH) questionnaire [58]. However, we hypothesize that the relationship between personality is stronger for multidimensional outcome measures that take psychosocial functioning into consideration as well (i.e., HS and (HR)QOL measures) [20]. How satisfied patients are with their functioning (HR)QOL, in contrast to an assessment of functioning (HS), might be particularly influenced by enduring patterns in behavior, cognition, and emotion that is labeled personality [59].

Pain catastrophizing is an important health cognition to take into account when treating DRF and AF. Higher pain catastrophizing was found in trajectories representing lower QOL (i.e., all QOL domains and the overall QOL facet). Those few studies that reported on pain catastrophizing in relation to HS/HRQOL in musculoskeletal trauma patients (e.g., fractures), indicated that pain catastrophizing is a significant predictor of HS/HRQOL five to eight months post-injury [28, 29]. Additionally, some studies focused on the relationship between pain catastrophizing and functional status after DRF [60–62]. Significant negative relationships were reported between pain catastrophizing and functional status 4 weeks [60] and 3 months after DRF surgery [61] whereas this association was not found in non-operatively treated patients with DRF 6 weeks post-fracture [62]. Our study sample encompassed approximately half DRF and half AF. More than two-third of the patients was non-operatively treated, suggesting that pain catastrophizing is important for QOL in the whole group of patients with either DRF or AF.

A more prominent role for internal PLOC (i.e., significance for all QOL domains and the overall QOL facet) was found compared to external PLOC (i.e., significance for overall, physical, and environmental QOL). Therefore, the belief of being in control of one’s own health seems a powerful cognition with positive relationships with QOL. The direction of this relationship is in agreement with research reporting positive associations between internal HLOC and the level of daily living activities/self-rated health in patients with hip fractures or patients at risk for cardiovascular disease [34, 63]. In contrast to pain catastrophizing and PLOC, patients did not differ on pain coping between QOL classes. We expected a significantly higher variability of pain coping strategies in patients for the trajectories representing higher QOL. However, variability does not directly imply an effective employment of these strategies that may explain the lack of association with QOL. Therefore, this is a limitation of the pain coping subscale that was used [33]. A suggestion might be to incorporate pain coping in further research using a somewhat different approach by examining more specific forms of coping in relation to QOL in patients with fractures (i.e., instead of variability). For example, to examine the suitability of passive versus active coping [30] or possible differences between problem focused, emotional or avoidance coping in relation to QOL [31].

Another possible limitation of our study includes the retrospective measurement of patients’ pre-injury QOL, which may have introduced recall biases. An inflation of pre-injury QOL may occur by re-evaluating pre-injury QOL with reference to the injured status (i.e., response shift). It was found that retrospectively reported pre-injury scores of HS and HRQOL were consistently higher compared to population norms [64]. However, the QOL measurements three, six, and 12 months post-fracture are assumed to be completed with the same internal standard, with reference to the injured status, as Time-0_retrospective_. Therefore, although the possibility of a small upward bias should be considered, the usage of retrospectively measured pre-injury QOL may be more appropriate than general population norms [64, 65].

Latent class trajectory analysis was used as a technique to gather new insights in complex QOL data of patients with fractures. Therefore, our paper presents a pragmatic application of this statistical taxonomic method. We do not claim that the clusters that were derived present the real theoretical clustering. However, using this taxonomy, we found different QOL trajectories that were defined by several sociodemographic, clinical, and psychological characteristics. These new insights could eventually contribute to the possible identification of patients at risk in clinical practice. The program Latent GOLD and the technique latent class trajectory analysis has already been used in other research areas in a similar pragmatic manner (i.e., as an exploring technique to obtain new insights). For example, in the field of cardiology [66–68] and perinatology [69]. Latent GOLD produces the most optimal taxonomy based on the data that have been gathered. With a small sample, replication with the use of a new dataset could show a different taxonomy. However, our sample size (*n* = 543) is relatively large. Therefore, we can be more confident that the results are stable and replicable. The total variance (*R* ^2^) explained by our models ranged from 71.6 to 79.4%, which means that individual variation between patients and over time is predicted very well by the classes. In addition, possible bias was taken into account at forehand using the Step 3 method.

This study attempts to encourage clinicians to take a biopsychosocial perspective in the treatment of patients with AF and DRF. Firstly, knowledge of several sociodemographic characteristics of patients is already informative regarding their course of QOL after fracture. However, differentiation is important. In general, the characteristics are different depending on the QOL domain of interest. For example, trajectories of social QOL did only differ significantly on marital and employment status and not on age, sex, or educational level. However, age was significant for overall, physical, psychological, and environmental QOL showing a broad scope of influence. Secondly, results suggested that it is important to be alert towards chronic comorbidities, especially for patients’ physical QOL. The presence of chronic comorbidities seems more crucial than several injury-related clinical variables. Thirdly, this was the first study in patients with DRF and AF including several psychological characteristics, showing that trait anxiety, neuroticism, extraversion, pain catastrophizing, and internal PLOC are significantly different between QOL trajectories (i.e., all QOL domains and the facet). Based on these results, further QOL research is recommended in which psychological predictors (e.g., pain anxiety, general illness beliefs [60, 62, 70]) in addition to sociodemographic and clinical indicators are incorporated. Based on the identified characteristics related to QOL by this study and further research using the biopsychosocial model, patients at risk for low QOL after fracture may be recognized earlier by health care professionals and, therefore, could be better monitored. A more personalized approach can be used, for instance when patients are severely hampered by negative pain beliefs. They may be offered additional care in the form of a psychological intervention aimed to lower pain catastrophizing [71].
